# Rectangular-Shaped Hemostatic Sutures in the Management of Second-Trimester Placenta Accreta Spectrum Disorders at Tu Du Hospital, Vietnam: A Retrospective Descriptive Study

**DOI:** 10.1055/a-2608-0990

**Published:** 2025-06-10

**Authors:** Van Hoang Bui, Hien Thi Nguyen, Le Quyen Nguyen, Phuong Thao Thi Truong, Phuong Thao Nguyen, Lam Phuong Thi Hoang, Phuc Nhon Nguyen

**Affiliations:** 1 Integrated Planning Room, Tu Du Hospital, Ho Chi Minh City, Vietnam; 2 Clinical Research Center (CRC), Tu Du Hospital, Ho Chi Minh City, Vietnam; 3 Department of Obstetrics and Gynecology, Pham Ngoc Thach University of Medicine, Ho Chi Minh City, Vietnam; 4 Department of Pregnancy Pathology, Tu Du Hospital, Ho Chi Minh City, Vietnam

**Keywords:** cesarean delivery, conservative surgery, complicated pregnancy, postpartum hemorrhage, placenta accreta spectrum, placenta previa, second trimester, ultrasound, Vietnam

## Abstract

**Objectives:**

The study aimed to delineate the surgical outcomes of rectangular-shaped sutures in PAS surgery.

**Materials and Methods** This retrospective study was conducted between January 2018 and December 2022 at Tu Du Hospital in Vietnam. The study reviewed all PAS cases below 22 weeks of gestational age (GA) that underwent cesarean delivery with rectangular-shaped hemostatic sutures. All the pregnancy characteristics, surgical features, and postoperative outcomes were described.

**Results:**

Among thirteen pregnant women with PAS, GA from 13 to 17 weeks of GA occupied 11/13 cases. PAS was classified as accreta ( *n* = 1), increta ( *n* = 1), increta-percreta ( *n* = 2), percreta ( *n* = 4), and percreta invasive to other organs ( *n* = 5). The estimated blood loss was 761.54 ± 614.12 (150–2,100 mL). Intraoperative blood loss between 500 and 1,500 mL accounted for 46.15%. The surgical duration time was 180.77 ± 32.07 (130–260 minutes). Postoperative duration time was 5.85 ± 2.08 (4–12 days). During the postpartum course, one case was reported with postpartum hemorrhage, acute renal dysfunction, and postoperative infection, respectively. Out of 13 PAS cases, 12 cases were successfully managed with conservative surgery.

**Conclusion:**

Surgical management of PAS disorders using rectangular-shaped hemostatic sutures could be acceptable. The technical suture is simple, safe, and cost-effective.

Placenta accreta spectrum (PAS), formerly known as morbidly adherent placenta, is defined as abnormal adherence of the placental trophoblast to the uterine myometrium. 1 PAS has risen dramatically due to the increasing rate of cesarean delivery. 2 3 Seriously, PAS is related to potentially life-threatening conditions for both mother and neonate, especially, in emergent conditions without adequate preparation. 4 5 Until today, many imaging modalities play an important role in the detection of PAS. 6 The accurate diagnosis could be performed in the first trimester. 7 8 A routine transvaginal ultrasound assessing a lower uterine segment scarred by previous cesarean section and placenta at 11 ^0/7^ and 13 ^6/7^ weeks of gestational age (GA) is a feasible and effective tool to identify significantly the risk of subsequent development of PAS disorders. 9

Importantly, early management involving planned surgery could reduce the mortality for pregnant women. 10 Currently, many surgical methods have been applied worldwide. 11 12 13 14 However, the management of second-trimester PAS is currently center-dependent with minimal evidence-based practices. Generally, hysterectomy remains a surgical option. 15 The surgical performance regarding GA below 22 weeks remains limited. Notably, the uterine artery embolization and left placenta in situ could not be applied in low-middle-income countries. Since the low-resource settings and lack of out-patient follow-up, the one-step surgical method is often chosen. In addition, the PAS should be managed at the expertise center with a multidisciplinary team. 16 17 18

Tu Du Hospital is a tertiary referral hospital in the south of Vietnam where PAS has been managed by an experienced team. In conservative surgery of PAS in the second trimester, the team applied the rectangular-shaped hemostatic suture prior to uterine incision to reduce the blood loss. This technique was developed by an obstetrician at our hospital (VHB). The team has made a finding on literature databases including Google Scholar, Medline, PubMed, Scopus, ScieLo, and Web of Science…; however, we did not find a similar suture in PAS surgical management. Through this study, we aim to describe the pregnancy outcomes of pregnancies diagnosed with PAS below 22 weeks undergoing the rectangular-shaped hemostatic suture during PAS surgery.

## Materials and Methods

### Study Design and Population

A retrospective descriptive study was conducted at Tu Du Hospital, Vietnam, between January 1, 2018 and December 31, 2022. The study included all pregnancies under 22 weeks GA which was diagnosed with PAS disorder following International Classification of Diseases 10th Revision (ICD-10) code O43.2. GA was calculated following the first-trimester ultrasound. In addition, the patient was managed with rectangular-shaped hemostatic surgery. This study was accepted by the ethical committee of the institution with approval number CS/TD/23/15.

Inclusion criteria: PAS disorder was diagnosed by ultrasound before surgery and at intraoperation by the surgeon's macroscopic observation. The histopathological examination was added to confirm the PAS. The patient underwent elective surgery or emergency surgery for cesarean delivery, receiving rectangular-shaped hemostatic surgery.

Exclusion criteria: Missing file, disorders of coagulation profile.

### Rectangular-Shaped Hemostatic Suture

**Video 1** Uterine incision following the rectangular-shaped hemostatic suture.


**Fig. 1:**
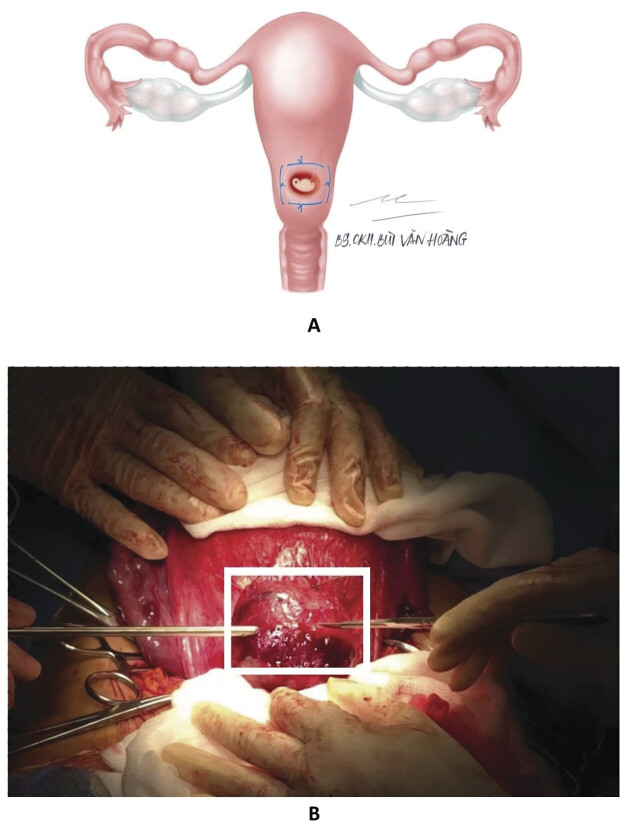
The rectangular-shaped hemostatic suture by illumination image ( **A** ) and intraoperative photo ( **B** ).

First, localization of the placental site by intraoperative ultrasound and macroscopic observation was determined at laparotomy. Second, the procedure of amniotic withdrawal under ultrasound was performed to reduce the size of the gravid uterus and facilitate uterine exteriorization. Third, suturing the marginal border of the placenta at the invasive myometrial layer of the uterus by using Chromic 1/0 absorbable suture. Likely, the suture line is similar to a rectangular shape ( Fig. 1A ). This suture was performed carefully before the uterine incision without uterine artery balloons or other flow-reducing devices ( Video 1 ). It is noteworthy that all the needling points for our patients were located in the avascular zone. After the suture is completely tightened, the neovascularization will be blocked. Therefore, the purpose of this technique was to reduce the bleeding following uterine incision. Even, the placenta was located at the posterior site or lateral site. Finally, the uterine incision was made through the placenta ( Fig. 1B ). The suture was removed with placental delivery and invasive myometrial resection if required. This suturing needle could touch the fetus during the procedure; thus, it should be applied in the condition of fetal abortion in PAS surgery.

### Surgical Management of Placenta Accreta Spectrum

**Video 2** A presentation of surgery technique relating to the management of placenta accreta spectrum less than 22 weeks of gestational age.


All the PAS cases under 22 weeks of GA were managed by a multidisciplinary team including an expert sonographer, anesthetist, and obstetrician. Before surgery, an ultrasound was performed to assess the PAS. The ultrasound criteria for diagnosis of PAS were performed according to The International Society of Ultrasound in Obstetrics and Gynecology (ISUOG) proposal. 19 20 The ultrasound was carried out by an abdominal or transvaginal transducer probe. Magnetic resonance imaging was indicated depending on the decision of clinicians. Cesarean delivery was performed under general anesthesia. Upon laparotomy, the PAS evaluation was observed carefully. Rectangular-shaped hemostatic suture was applied. After the uterine incision, all placental tissue was delivered completely. Other hemostatic sutures could be added if necessary. Additionally, oxytocin 10 UI was given after placental delivery. Uterotonic drugs such as ergometrine, Duratocin, and hemostatic drug (acid tranexamic) were applied when necessary. The uterine myometrial reconstruction was performed. The vaginal bleeding was checked before closure of the abdomen. One surgeon with more than 15 years of experience in the obstetrical field performed this suture ( Video 2 ).

### Data Collection

All the information was collected based on the patient's file.

**Fig. 2:**
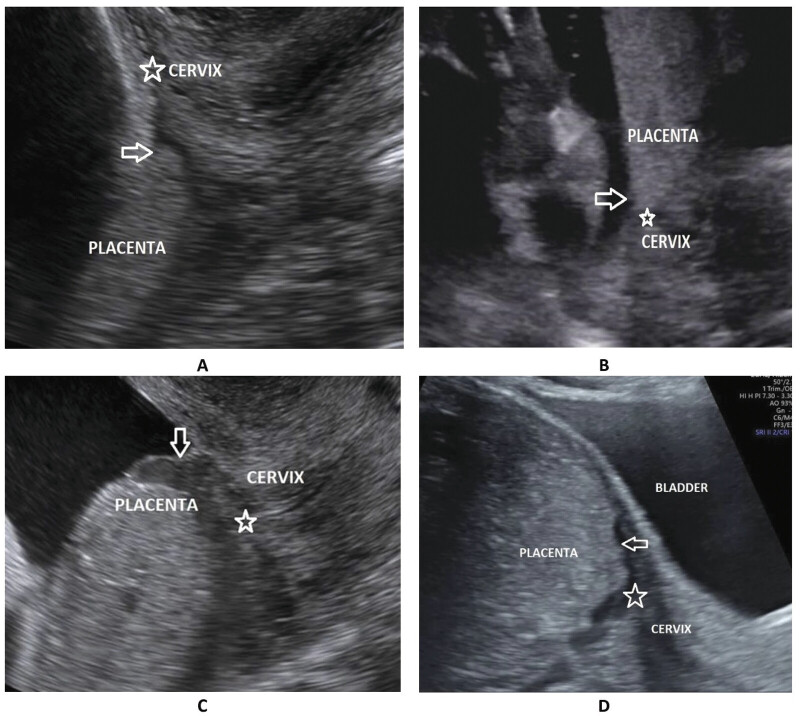
The classification of placenta previa depends on the distance between the lower edge of the placenta (white arrow) and the internal cervical os (white star). This classification on ultrasound includes type I, low-lying placenta ( **A** ), type II, marginal placenta ( **B** ); type III, incomplete/partial central placenta ( **C** ); and type IV, complete/central placenta ( **D** ).

**Fig. 3:**
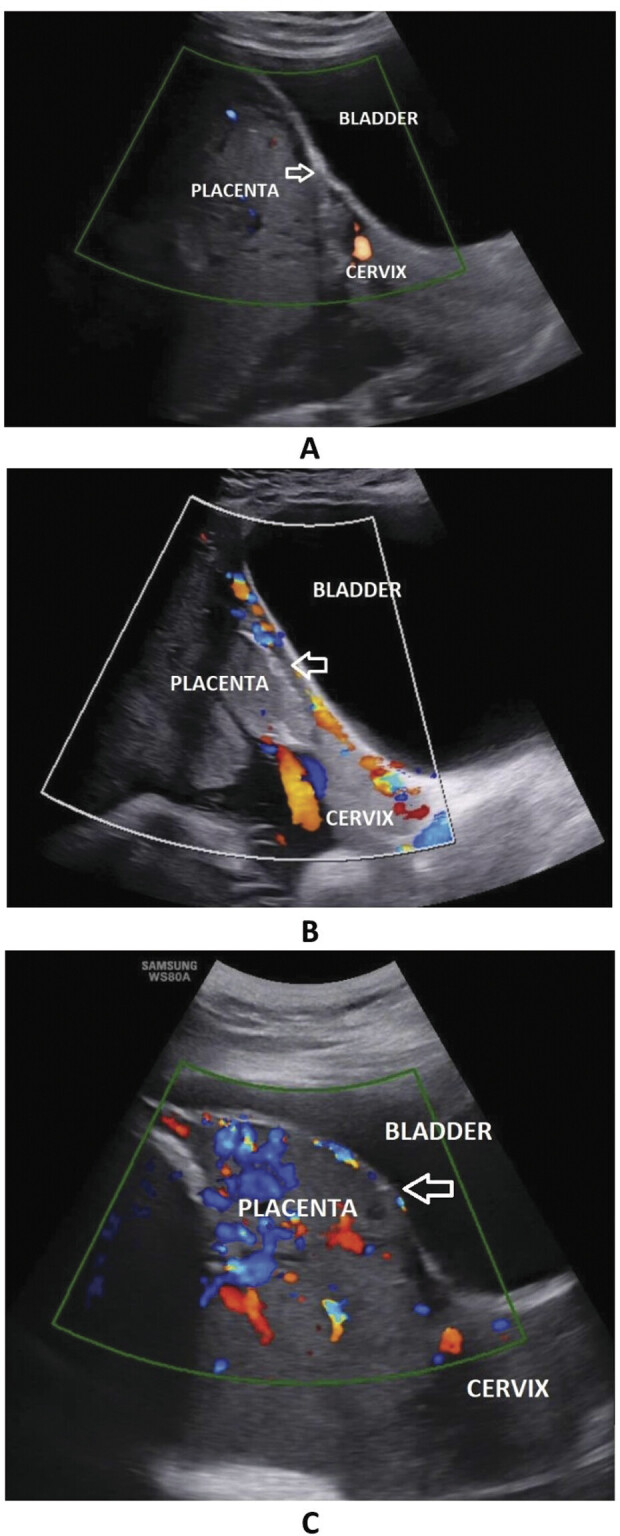
Ultrasound images show the myometrial thickness, vesicouterine interface (white arrow), railway sign, lacunae, placental budge, and the degree of hypervascularity on color Doppler ultrasound. According to imaging characteristics, the types of placenta accreta spectrum disorders are classified as accreta ( **A** ), increta ( **B** ), and percreta ( **C** ).

– Continuous variables: Maternal age (years), GA (weeks), body mass index (kg/m ^2^ ), intraoperative estimated blood loss (mL), operation duration time (minutes), postpartum course (days).
– Categorical variables: Types of the surgical method, previous cesarean scar, type of placenta previa on ultrasound ( Fig. 2 ), bilateral uterine arteries ligation, other hemostatic procedures, left partial placenta in situ, ureteral injury, operation duration time, postoperative hemorrhage, and postoperative infection. The grade of PAS disorders according to the International Federation of Gynecology and Obstetrics (FIGO) 2018 ( Fig. 3 ). 21

### Statistical Analysis

Data were statistically analyzed by Statistical Package for the Social Sciences (SPSS) version 22.0 (SPSS Inc, Chicago, Illinois). Frequency ( *n* ), percentage (%), mean ± standard deviation (SD), median, and interquartile range were used as measurement data according to the distribution of data.

## Results

In our study, thirteen cases were eligible for the study. Among them, the GA from 13 to 17 weeks of GA occupied 11/13 cases. Ten cases were asymptomatic ( Table 1 ).

**Table 1:** Baseline characteristics of the study population

Characteristics		Data
Maternal age (y)		33.23 ± 3.35 (27–38)
Body mass index (kg/m ^2^ )		21.87 ± 2.90 (19.53–29.97)
Gravida (times)	2	5 (38.4)
	3	4 (30.8)
	≥4	4 (30.8)
Prior cesarean scar (times)	1	3 (23.1)
	2	8 (51.5)
	≥3	2 (15.4)
History	Surgery of uterine corpus	1 (7.7)
	Abdominal surgery	13 (100.0)
	Uterine malformation a	1 (7.7)
	Uterine fibroid	1 (7.7)
	Cesarean scar pregnancy	1 (7.7)
	PAS	0 (100.0)
Time interval between two pregnancies	>12 mo	12 (92.3)
Clinical symptoms	Asymptomatic	10 (76.9)
	Vaginal bleeding/abdominal pain	3 (23.1)
Gestational age (wk)	13–14	6 (46.2)
	14–15	1 (7.7)
	16–17	4 (30.8)
	18–19	1 (7.7)
	20–21	1 (7.7)
Alive fetus		13 (100.0)

Note: Data was presented as *n* (%) and mean ± SD (min–max).

a Bicornuate uterus.

Table 2 shows the ultrasonic features of PAS. More than half of cases are located at the anterior wall of the uterus and classified as placenta previa type III–IV. Nine cases were diagnosed with PAS-type percreta. The remained myometrial thickness less than 1 mm was observed in 10 cases. Commonly, the newly vessel proliferation was detected in 8 cases. In addition, the mean hemoglobin of the pregnant women before surgery was 11.54 ± 0.86 (g/dL).

**Table 2:** Paraclinical characteristics of PAS cases under 22 weeks of gestational age

Characteristics		Data
Hemoglobin (g/dL)	Mean ± SD (min–max)	11.54 ± 0.86 (9.9–13.1)
Hematocrit (%)	Mean ± SD (min–max)	34.16 ± 2.65 (28.3–38.8)
Placental site	Anterior	6 (46.1)
	Posterior	3 (23.1)
	Anterio-posterior	3 (23.1)
	Lateral	0 (0.0)
	Left latero-posterior	1 (7.7)
Type of placenta previa	I	0 (0.0)
	II	0 (0.0)
	III	6 (46.2)
	IV	7 (53.8)
Grade of PAS	Accreta	0 (0.0)
	Increta	3 (23.1)
	Percreta	2 (15.3)
	Accreta-increta	1 (7.7)
	Increta-percreta	3 (23.1)
	Percreta invaded other organs	4 (30.8)
Grading of intraplacental lacunae	1	4 (30.8)
	2	6 (46.1)
	3	0 (0.0)
	Not evaluated	3 (23.1)
Loss of “clear zone”	Yes	3 (23.1)
	No	7 (53.8)
	Not evaluated	3 (23.1)
Myometrial thinning (<1 mm or undetectable)	Yes	10 (77.0)
	No	1 (7.7)
	Not evaluated	2 (15.3)
Neovascularization signs	Absent/mild	0 (0.0)
	Moderate	2 (15.3)
	Tortuous	8 (61.6)
	Not evaluated	3 (23.1)
Abnormal uterine margin/borderline	Yes	3 (23.1)
	No	8 (61.6)
	Not evaluated	2 (15.3)
Focal exophytic mass or placental bulging	Yes	5 (38.4)
	No	6 (46.2)
	Not evaluated	2 (15.4)
Bridge vessels	Yes	2 (15.4)
	No	9 (69.2)
	Not evaluated	2 (15.4)
PAS invaded to pelvic organs	No	8 (61.5)
	Bladder	1 (7.7)
	Ureteral	0 (0.0)
	Pelvic wall	0 (0.0)
	Cervix	0 (0.0)
	More than 2 organs	2 (15.3)

Note: Data was presented as *n* (%) and mean ± SD (min–max).

In this study, almost cases were scheduled for planned surgery, except for 3 cases receiving emergent surgery due to vaginal bleeding. In surgery, PAS was classified as accreta ( *n* = 1), increta ( *n* = 1), increta-percreta ( *n* = 2), percreta ( *n* = 4), and percreta invasive to other organs ( *n* = 5). In this study population, bilateral uterine artery ligation of the superior branch was additionally applied in all cases. Approximately, three-quarters of cases required placenta bed sutures and ligation of uterine branches originating from the ovarian artery. The estimated blood loss was 761.54 ± 614.12 (150–2,100 mL). Intraoperatively, the estimated blood loss from 500 to 1,500 mL occupied 46.15% of cases. Among PAS types, PAS type percreta relates to severe blood loss (1,210.0 ± 610.7 mL). The surgical duration time was 180.77 ± 32.07 (130–260 minutes). The postoperative duration time was 5.85 ± 2.08 (4–12 days; Table 3 ).

**Table 3:** Intraoperative features of PAS management below 22 weeks of gestational age

Characteristics		Data
Grade of PAS	Accreta	1 (7.7)
	Increta	1 (7.7)
	Percreta	4 (30.8)
	Accreta-increta	0 (0.0)
	Increta-percreta	2 (15.4)
	Percreta invaded other organs	5 (38.5)
Placental site	Bilateral	1 (7.7)
	Left lateral	0 (0.0)
	Right lateral	1 (7.7)
	Anterior and posterior	4 (30.8)
	Posterior	1 (7.7)
	Anterior	6 (50.0)
Skin incision	Sub-umbilical midline	2 (15.4)
	Pfannenstiel	11 (84.6)
Myometrial restoration	Yes	11 (84.6)
	No	1 (7.7)
	Not recorded	1 (7.7)
Left placenta in situ	Yes	0 (0.0)
	No	13 (100.0)
Type of surgery	Planned surgery	10 (76.92)
	Emergent surgery	3 (23.08)
Surgical method	Hysterectomy	1 (8.33)
	Conservative surgery	12 (91.67)
Hemostatic suture besides rectangular-shaped suture	Bilateral uterine artery ligation (superior branch)	13 (100.0)
	Uterine branch of the ovarian artery	10 (77.0)
	Placental bed	10 (77.0)
	Transverse and vertical B-lynch compression	2 (15.4)
	Bilateral cervical uterine artery ligation + bilateral uterine artery ligation (inferior branches) + intrauterine balloon insertion	1 (7.7)
Intraoperative estimated blood loss (mL)	Mean ± SD (min–max)	761.54 ± 614.12 (150–2,100)
	<500	4 (30.77)
	500–1,500	6 (46.15)
	1,500–2,000	2 (15.39)
	>2,000	1 (7.69)
Intraoperative estimated blood loss (mL) following types of PAS	Accreta	337.5 ± 187.5
	Increta	625.0 ± 613.1
	Percreta	1,210.0 ± 610.7
Ureteral/vesical injuries	Yes	0 (0.0)
	No	13 (100.0)
Surgical duration time (min)	Mean ± SD (min–max)	180.77 ± 32.07 (130–260)

Note: Data was presented as *n* (%) and mean ± SD (min–max).

During the postpartum course, one case was noted with postpartum hemorrhage, one case was noted with acute renal dysfunction, and one case was noted with postoperative infection. Out of 13 PAS cases, 12 cases were successfully managed with conservative surgery. No maternal death was reported ( Table 4 ).

**Table 4:** Surgical outcomes in the present study

Characteristics		Data
Postoperative duration time (d)	Mean ± SD (min–max)	5.85 ± 2.08 4 5 6 7 8 9 10 11 12
Complications	Postpartum hemorrhage	1 (7.7)
	Intraabdominal bleeding	0 (0.0)
	Acute renal dysfunction	1 (7.7)
	DIC	0 (0.0)
	Postpartum infection	1 (7.7)
Antibiotic therapy	1 group	1 (7.7)
	≥2 group	12 (92.3)
Maternal death	Yes	0 (0.0)
	No	13 (100.0)
Histopathological examination a	Increta	1 (7.7)
	Percreta	8 (61.5)
	Unidentified PAS	1 (7.7)

Note: Data was presented as *n* (%) and mean ± SD (min–max).

a Two cases were not sent for histopathological examination and one case was not recorded in the patient's file.

## Discussion

Globally, a lot of practical methods have been applied in second-trimester PAS management such as vaginal delivery, leaving the placenta in situ by combining methotrexate, elective artery embolization, one-step surgical approach, and cesarean hysterectomy by laparotomy and laparoscopy. 22 23 24 However, embolization is expensive and may not increase the effectiveness of treatment. 25 Following Hu et al, terminating a pregnancy by vaginal delivery through medical induction of labor may be feasible if clinicians have an overall understanding of GA, the type of placenta previa status, the type of placenta accreta, and patients' concerns about preserving fertility. 26 Conversely, we decided to perform one-step conservative surgery with the removal of the placenta and restoration of uterine myometrium with an invasive placenta due to the local condition. Recently, Hessami et al have also demonstrated that conservative management for pregnancies with PAS is associated with reduced surgical morbidity and may offer an effective alternative to cesarean hysterectomy. 27

Additionally, the hemostatic suture was used during PAS surgery. Regarding PAS in the second trimester, we used the rectangular-shaped suture to reduce blood loss before uterine incision and fetal delivery. Before surgery, the team investigated the placental characteristics with ultrasound assessment to determine the grade of PAS and the location of the placenta. According to Panaiotova et al, accurate prediction of PAS can be achieved by ultrasound examination at 12 to 16 weeks gestation of the pregnancy with previous uterine surgery and low-lying placenta. 28 A finding of the placenta under or within the scar niche should prompt further assessment at a PAS-specialized center. 29 30 In the present study, almost all cases had at least one cesarean scar and were classified as placenta previa type III–IV. At our tertiary referral hospital, the sonographic assessment found four cases of type increta and nine cases of type percreta. Intraoperatively, the team identified one PAS type accreta, one PAS type increta, and eleven cases type percreta. Among them, 10 cases had surgical specimens for histopathological examination. Histology showed one case type increta, eight cases type percreta, and one unidentified case.

In our study, the estimated blood loss was 761.54 ± 614.12 mL. In a study by Li et al, cesarean delivery on 28 PAS cases in the second trimester related to increased blood loss (932.14 ± 940.86 mL). 25 In our study, 4/13 cases (30.8%) of PAS underwent emergent cesarean delivery due to vaginal bleeding and abdominal pain. In line with Luccidi et al, emergency cesarean delivery complicates approximately 35% of pregnancies affected by PAS disorders and is associated with a higher risk of adverse maternal and neonatal outcomes. 31 Hysterectomy was noted in 21/51 cases in the study of Hu et al. Among 21 cases of hysterectomy, 17 cases were more than 17 weeks of GA. The blood loss was 752.38 ± 1,310.58 mL. 26 To achieve hemostasis, some drugs such as tranexamic acid could be added. 5 Ideally, a collaborative team effort in tertiary medical centers with a very experienced multidisciplinary team and combined application of multiple methods is required to facilitate patient outcomes. 26 Protocol-based interdisciplinary care from diagnosis to surgery will optimize both intraoperative and postoperative outcomes. 32

### Strengths and Limitations

To our knowledge, this study is the first report concerning the rectangular-shaped hemostatic suture. The technique was performed by the same surgeon. However, this study included a small sample size. Due to ethical concerns, the study could not carry out a prospective study with a control group (without rectangular-shaped hemostatic suture). The retrospective could not avoid the recall bias. The PAS surgery included other hemostatic procedures; thus, the role of rectangular-shaped hemostatic suture was difficult to evaluate fully. Furthermore, long-term outcomes on menstrual status and subsequent pregnancies have not yet been investigated.

## Conclusion

In summary, rectangular-shaped hemostatic sutures could be an easy, cheap, and efficient surgical choice in PAS surgery for pregnancies under 22 weeks of GA. It can greatly minimize the amount of blood loss and reduce the risk of hysterectomies and maternal mortality. Further well-designed studies with large samples are warranted to confirm our findings.
